# Moderate nutritional stress reprogrammes insulin responses to drive enhanced starvation tolerance in *Drosophila melanogaster*

**DOI:** 10.1242/jeb.250507

**Published:** 2026-01-26

**Authors:** Reshma V. Menon, Jishy Varghese

**Affiliations:** School of Biology, Indian Institute of Science Education and Research (IISER TVM), Thiruvananthapuram 695551, Kerala, India

**Keywords:** *Drosophila melanogaster*, Insulin signalling, Metabolic adaptation, Starvation stress, Time-restricted feeding, Enhanced starvation resilience

## Abstract

Organisms in the wild constantly encounter fluctuations in temperature and food availability, pathogens and other stressors that disrupt their physiological balance. To counteract these disruptions, organisms initiate stress responses that vary in nature depending on the intensity and duration of the stressor. While severe stress can be harmful or even fatal, moderate stress can activate adaptive mechanisms, a phenomenon known as hormesis. Hormesis enhances resilience to stress and has been associated with improved ageing, immunity and metabolism. Short-term exposures to mild stress, such as heat or oxidative stress, have been shown to extend *Drosophila* lifespan and promote cross-tolerance to other stressors. Among various environmental stressors, starvation poses a significant and recurring challenge that has driven the evolution of energy-conserving strategies essential for survival. Prior exposure to starvation has been shown to influence longevity, resilience to starvation, physiological status and stress tolerance. However, the mechanisms underlying these hormetic effects remain poorly understood. In this study, we investigated how short-term starvation enhances resistance to prolonged food deprivation in *Drosophila*. Our findings reveal that metabolic rewiring, including changes in energy utilization, insulin signalling and transcriptomic profiles, underpins this adaptive plasticity. These insights will improve our understanding of the molecular and metabolic mechanisms driving hormesis, with broader implications for stress resilience and organismal health.

## Teaser

**Summary:** Brief periods of starvation stress aid in a hormetic reprogramming of metabolism and starvation-induced insulin responses in *Drosophila melanogaster* and confer enhanced starvation tolerance on them.

## INTRODUCTION

The state of constancy is a luxury rarely afforded to organisms in the wild. Environmental conditions frequently fluctuate, with variations in temperature, humidity, food and water availability, and exposure to pathogens. These external changes disrupt an organism's physiological equilibrium, triggering stress responses aimed at restoring balance (Cannon, 1935). The nature of these responses depends largely on the intensity and duration of the stressors. While severe stressors can cause irreversible damage and may even be lethal, moderate stress levels activate adaptive homeostatic mechanisms that help maintain the organism's internal stability (Selye, 1975; Cannon, 1935; Holmes, 1986). However, there remains a significant gap in our understanding of how organisms respond and adapt to sustained stress.

Hormesis involves adaptive changes following exposure to a stressor, enhancing the organism's ability to withstand similar threats in the future (Schulz, 1887, 1888; Southam et al., 1943). Hormesis has been implicated in various aspects of biology, including ageing, immunology, cancer biology and metabolism (Calabrese, 2005, 2013; Gems and Partridge, 2008; Le Bourg, 2011, 2020; Jun et al., 2017; Zhou et al., 2018). Experimental investigation of hormesis has revealed the complex relationship between stress and stress resilience. The fruit fly *Drosophila melanogaster* has become a prominent model organism in hormetic biology because of its ease of handling and the ability to administer a wide range of stressors. Research has shown that short-term stress exposure can significantly impact the physiology of *Drosophila*. Various stressors, including mild heat shock, larval crowding, hypergravity, cold and oxidative stress, have been associated with lifespan extension (Le Bourg et al., 2001; Hercus et al., 2003; Baldal et al., 2005; Le Bourg, 2007; Ristow and Schmeisser, 2011). Additionally, prior exposure to stress has been linked to hormetic cross-tolerance, where flies demonstrate enhanced resilience to different stressors. This underscores the adaptive benefits of stress exposure (Le Bourg, 2007, 2016; Le Bourg et al., 2009; Pickering et al., 2013; Parkash et al., 2014; Henry et al., 2018; Bomble and Nath, 2019).

Among metabolic stressors, starvation stands out as a significant and recurring challenge faced by organisms. The persistence of this threat has driven the evolution of adaptive strategies to counteract stress and maintain energy homeostasis during food deprivation. Research has demonstrated that prior exposure to starvation can significantly influence various biological outcomes, including food preference, longevity, heart function and resilience to other stressors (Turelli and Hoffmann, 1988; Partridge et al., 2005; Kezos et al., 2017; De Ro et al., 2021). Previously, we reported that caloric restriction during the larval stage affects adult phenotypes, such as increased starvation resistance and altered metabolite levels, by altering expression of the lipase gene *brummer* (*bmm*) and the perilipin gene *lipid storage droplet 2* (Rehman and Varghese, 2021). Similarly, mild starvation can be induced in flies by restricting their access to food through time-restricted feeding (TRF). TRF has been shown to improve muscle function through the AMPK signalling pathway and the purine cycle (Villanueva et al., 2019; Livelo et al., 2023). Additionally, TRF mitigates age-related cardiac dysfunction by regulating the circadian clock, the TCP-1 ring complex (TRiC) chaperonin and the mitochondrial electron transport chain (Gill et al., 2015). Furthermore, TRF reverses high-fat diet-induced metabolic dysregulation by fine tuning the peripheral clock machinery (Salgado-Canales et al., 2023). While the hormetic benefits of dietary restriction in enhancing resistance to prolonged starvation are well documented (Chippindale et al., 1993; Bubliy et al., 2012; Katewa et al., 2016; Lenhart et al., 2024; Catterson et al., 2018), the underlying mechanisms and associated metabolic rewiring remain poorly understood. Investigating these processes can provide valuable insights into the dynamic biological adaptations triggered by nutritional stress and their implications for health and disease.

In this study, we demonstrate that brief episodes of starvation stress enhance resistance to prolonged starvation in flies, providing strong evidence for hormesis. Early starvation exposure induces tolerance to oxidative and heat stress, indicating cross-tolerance in trained flies. The heightened resistance is achieved through extensive metabolic rewiring, including alterations in energy storage and utilization during chronic starvation. Additionally, trained flies exhibit reduced activity, probably contributing to energy conservation and improved survival. Notably, we identify whole-body transcriptomic changes linked to hormesis, offering key insights into the molecular mechanisms underlying stress-induced adaptive plasticity. Furthermore, our findings underscore the pivotal role of the insulin signalling pathway in establishing and maintaining starvation tolerance in stress-exposed flies.

## MATERIALS AND METHODS

### Fly strains and husbandry

*Drosophila melanogaster* flies were reared in vials with standard cornmeal media containing 5.8% cornmeal, 5% dextrose, 2.36% yeast, 0.8% agar and 10% nipagin dissolved in 100% ethanol on a 12 h:12 h light:dark cycle in humidity- (70% relative humidity) and temperature- (25°C) controlled incubators (Percival Scientific, Perry, IA, USA) (unless specified otherwise for specific experiments; see next paragraph). The fly strains *w^1118^* (RRID:BDSC_3605) and *s_1_106*-GAL4 (RRID:BDSC_8151) were obtained from Bloomington Stock Centre (BDSC, Indiana University, USA)*. inr-RNAi* (#992) was procured from Vienna Drosophila Resource Centre (VDRC, Vienna, Austria). *dilp2-*GAL4-GS/CyO was a kind gift from Dr Marc Tatar (Brown University, USA). *dilp2*GAL4gfp/CyO; *tub*GAL80^ts^/TM2 line was a generous gift from Dr Stephen M. Cohen (University of Copenhagen, Denmark). UAS*-kir2.1* line was kindly gifted by Dr Sheeba Vasu (Jawaharlal Nehru Centre for Advanced Scientific Research, India).

For temperature-switch experiments performed with the fly line *dilp2*GAL4gfp/CyO; *tub*GAL80^ts^/TM2, the flies were shifted to the permissive temperature of 29°C for the activation of GAL4 and to the restrictive temperature of 18°C for the inactivation of GAL4 as required. For experiments with GeneSwitch lines *dilp2-*GAL4-GS and *s_1_106*-GAL4, the flies were transferred to food vials containing 0.5 mg ml^−1^ mifepristone/RU486 (#M8046, Sigma-Aldrich, St Louis, MO, USA) for the activation of GAL4 as required. All experiments were performed on male flies that were randomly assigned to control or experimental groups. The sample size (*n*) was determined based on previous experiments conducted in the lab and multiple biological replicates were run to ensure robustness and reproducibility. All the samples post-training were processed for experiments.

### Starvation resistance assay

**Fig. 1.**
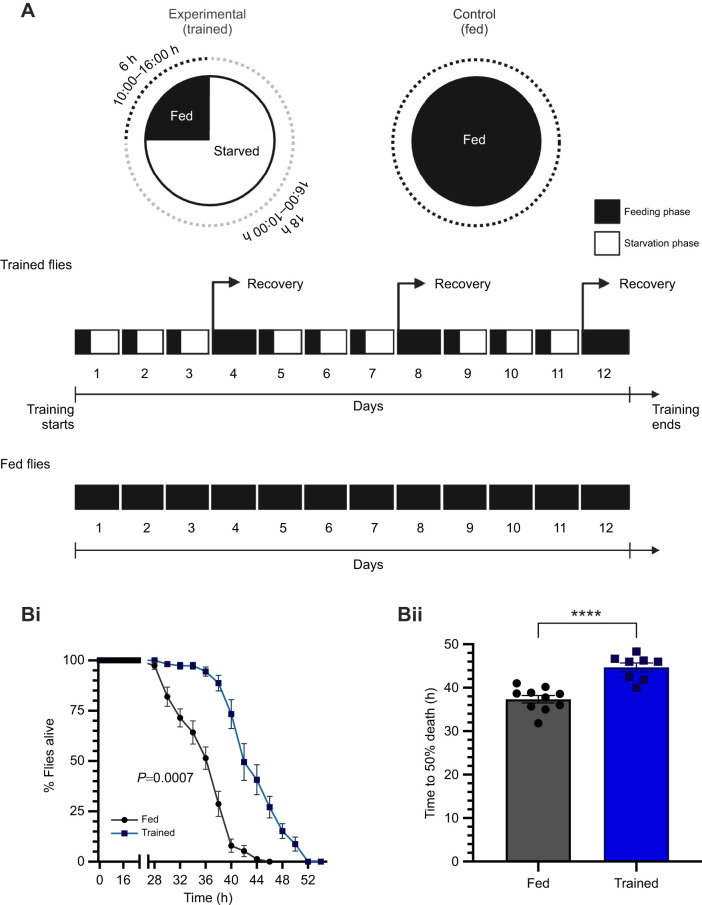
**Cyclical feeding–starvation regime establishes enhanced starvation resistance.** (A) The training regime. All experiments were performed on day 13 flies unless specified otherwise. (Bi) Survivorship curve of the fed and trained flies under chronic starvation (fed: *n=*10, trained: *n*=8; log-rank test; *P*=0.0007). (Bii) The median mortality of flies under chronic starvation (two-tailed Student's *t*-test with Welch's correction; *P*<0.0001). Data are presented as means±s.e.m.

A total of 50 first instar larvae were reared in vials containing standard cornmeal media. Once eclosed, 15 male flies were collected per vial and subjected to a 6 h:18 h feeding–starvation protocol (Fig. 1A) for a duration of 12 days for the trained set or kept in food vials constantly for the control set. Following the 12 day training period, the fed and trained flies were subjected to chronic starvation by transferring them to 1% agar vials. The number of dead flies was noted every 2 h. Multiple replicates were carried out to ensure robustness and reproducibility of the data. The percentage survival across time and median survival were plotted and analysed using GraphPad Prism. Survivorship curves were analysed with OASIS 2 software.

### Triglyceride and glycogen estimation assays

The experimental protocol for training was followed and the day 13 trained and fed flies were then subjected to starvation in 1% agar vials and samples were taken at 0, 16 and 28 h during starvation. Groups of 5 flies were placed into Eppendorf safe-lock tubes (#0030120086, Eppendorf, Hamburg, Germany). Homogenization was carried out in 400 µl of 0.05% Tween-20 using Bullet Blender Storm (#BBY24M, Next Advance, Troy, NY, USA). The lysate was subjected to heat inactivation at 70°C for 6 min followed by centrifugation at maximum speed for 3 min to sediment cellular debris. The supernatant was added to a 96-well plate and the triglyceride and glycogen levels were determined using a triglyceride quantification kit (#TR0100, Sigma-Aldrich) and a glycogen colorimetric quantification kit (#MAK016, Sigma-Aldrich), respectively, following the manufacturer's instructions. Protein levels were assessed in parallel using Quick Start Bradford Dye Reagent (#5000205, Bio-Rad, Hercules, CA, USA). The colorimetric readings were taken at 540 nm for triglyceride, 595 nm for protein and 570 nm for glycogen quantification using a TECAN Infinite M200 Pro Multimode plate reader (TECAN, Männedorf, Switzerland). The absorbance readings were noted and subsequently plotted using GraphPad Prism. Data from multiple replicates were taken to ensure robustness and reproducibility.

### Glucose estimation assay

The experimental protocol for training was followed as above, and the day 13 experimental and control flies were used for haemolymph collection. The thorax region of the flies was pricked using a fine needle followed by centrifugation in ZymoSpin columns (#C1006250, Zymo Research, Orange, CA, USA). The collected haemolymph was diluted 1:100 in molecular biology grade water (#ML024, HiMedia, Thane, Maharashtra, India) and processed for glucose estimation using a glucose assay kit (#GAGO20, Sigma-Aldrich) following the manufacturer's instructions. The resulting solution was plated into 96-well plates and colorimetric readings were taken at 540 nm using the TECAN Infinite M200 Pro Multimode plate reader. The absorbance readings were recorded and plotted using GraphPad Prism. Data from multiple replicates were taken to ensure robustness and reproducibility.

### Trehalose estimation assay

The experimental protocol for training was followed and the day 13 experimental and control flies were used for haemolymph collection. The protocol for haemolymph trehalose estimation was adapted from Tennessen et al. (2014).

Briefly, the thorax region of the flies was pricked using a fine needle followed by centrifugation in ZymoSpin columns (#C1006250, Zymo Research). The collected haemolymph was diluted 1:100 in trehalase buffer then subjected to heating at 70°C to carry out inactivation of endogenous trehalase enzyme. Subsequent aliquots of the samples were prepared with and without trehalase enzyme to account for the free glucose in the haemolymph and incubated overnight at 37°C. Following this, a glucose assay was performed (#GAGO20, Sigma-Aldrich) following the manufacturer's instructions. The resulting solution was plated into 96-well plates and colorimetric readings were taken at 540 nm using the TECAN Infinite M200 Pro Multimode plate reader. The absorbance readings were recorded and plotted using GraphPad Prism. Data from multiple replicates were taken to ensure robustness and reproducibility.

### Feeding assay

The experimental protocol for training was followed and the day 13 fed and trained flies (10 flies per vial) were transferred to agar vials containing yeast paste mixed with Orange G dye (#1936158, Sigma-Aldrich) and allowed to feed for 30 min. Groups of 5 flies were homogenized in 0.05% Tween-20 (#P9416, Sigma-Aldrich). The supernatant was added to 96-well plates and colorimetric readings were recorded at 478 nm using the TECAN Infinite M200 Pro Multimode plate reader.

For starvation-induced feeding assay, the flies were starved for 6 and 12 h prior to being introduced into the coloured food vials. The subsequent protocol was the same as for the *ad libitum* feeding assay.

### Oxidative stress resistance assay

The training protocol was followed and day 13 fed and trained flies (10–15 of each) were transferred to 1% agar vials for 3 h. These flies were then shifted to vials containing filter paper soaked in 5% sucrose with 20 mmol l^−1^ paraquat (#856177, Sigma-Aldrich). Every 12 h, the flies were flipped into vials containing a fresh filter paper with sucrose and paraquat. The number of flies that died was recorded. The data were plotted and analysed using GraphPad Prism.

### Desiccation stress resistance assay

Day 13 fed and trained flies (10–15 of each) were transferred to empty glass vials. The number of flies that died was recorded every 2 h. The data were plotted and analysed using GraphPad Prism.

### Activity–rest profile

The experimental protocol for training was followed and the day 13 experimental and control flies were individually housed in activity tubes. The locomotor activity was recorded using *Drosophila* Activity Monitors (DAM, Trikinetics, Waltham, MA, USA) under a 12 h:12 h light:dark cycle at 25°C. The DAM system detects bouts of activity as breaks in the infrared beam caused by the fly's movement. The recording of the activity–rest rhythm of the flies was done over 5 days during which they had constant access to food. The cumulative activity profile for 5 days was plotted and analysed using GraphPad Prism. Data from multiple replicates were taken to ensure robustness and reproducibility.

### Quantitative real-time PCR (qPCR)

The experimental protocol for training was followed and the day 13 trained and control fed flies (7 per replicate for whole-body qPCR and 30 heads per replicate for head qPCR) were transferred to 1% agar and flash frozen in liquid nitrogen at 0, 16 and 28 h of starvation. RNA extraction was carried out using TriZoL Reagent (#15596018, Invitrogen, Waltham, MA, USA) following the manufacturer's instructions and further processed using the phenol–chloroform method (detailed protocol from protocols.io, https://doi.org/10.17504/protocols.io.fgtbjwn). The resultant RNA was quantified using NanoDrop and normalized across samples before being converted to cDNA using PrimeScript™ RT Reagent Kit (#RR037B, Takara Bio Inc., Shiga, Japan) following the manufacturer's instructions. The resultant cDNA was used for qPCR using TB Green Premix Ex Taq (#RR82WR, Takara Bio Inc.). A list of primers is provided in Table S1A. Data were analysed using GraphPad Prism.

### Nile Red staining

Day 13 fed and trained flies were dissected to isolate the abdominal cuticle with attached fat body in ice-chilled Shields and Sang M3 Insect Medium (#S8398, Sigma-Aldrich). The tissue was fixed in 4% formaldehyde, washed twice using 1× phosphate-buffered saline (PBS; #P4417, Sigma-Aldrich) and incubated with Nile Red (#72485, Sigma-Aldrich) diluted 1 µg ml^−1^ in 75% glycerol for 30 min at room temperature. The samples were rinsed with Milli-Q ultrapure water and the fat tissue was mounted on a slide. Imaging was done using Zeiss LSM880 confocal microscope. The images were analysed with ImageJ and data analysis was performed with GraphPad Prism.

### Western blotting

Day 13 fed and trained flies (5 flies per replicate) were flash frozen in liquid nitrogen at 0, 16 and 28 h of starvation. Flies were homogenized in RIPA buffer containing protease inhibitor (#4693132001, Sigma-Aldrich) and phosphatase inhibitor (#P5726, Sigma-Aldrich). The samples were mixed with 2× Laemmli sample buffer (#1610737, Bio-Rad) in 1:1 ratio and denatured at 70°C. The cellular debris was pelleted and the supernatant was loaded and run in a 10% SDS-polyacrylamide gel. The proteins were blotted onto PVDF membrane, blocked and incubated with primary antibodies p-Akt (1:1000; #4060, Cell Signaling Technology, Danvers, MA, USA), t-Akt (1:1000; #9272, Cell Signaling Technology) and actin (1:3000; #612656, BD Biosciences, San Diego, CA, USA), followed by appropriate HRP-conjugated anti-rabbit (#7074, Cell Signaling Technology) and anti-mouse (#7076, Cell Signaling Technology) secondary antibodies. The antibody validation information from previous studies is listed in Table S1B. The proteins were visualized using Immobilon Western Chemiluminescent HRP Substrate (#WBKLS0050, Merck Millipore, Danvers, MA, USA) and the protein bands were quantified via ImageJ. Data were plotted and analysed with GraphPad Prism.

### Insulin sensitivity assay

Day 13 fed and trained ice-anaesthetized flies were dissected to isolate abdominal cuticle with attached fat body in ice-chilled Shields and Sang M3 Insect Medium. The dissected tissue was incubated in insect medium at room temperature for 15 min. The insect medium was replaced with either fresh insect medium or fresh insect medium containing 1 µmol l^−1^ of human insulin solution (#I9278, Sigma-Aldrich) and incubated for 15 min. Post-incubation, the solution was discarded and the resultant tissue was used to run western blots using the protocol described above.

### Enzyme linked immunosorbent assay (ELISA)

The experimental protocol for training was followed and the day 13 experimental and control flies were used for haemolymph collection. The thorax region of the flies was pricked using a fine needle followed by centrifugation in ZymoSpin columns (#C1006250, Zymo Research) at 4°C; 1 µl of the extracted haemolymph was diluted with 100 µl solution of 1× PBS and 50 µl of the diluted haemolymph was coated on the wells of a 96-well plate and incubated overnight at room temperature. Following this, the sample solution was removed and 300 µl of the blocking buffer BBT {0.1% BSA (#A2153, Sigma-Aldrich) in PBT [0.1% Triton X-100 (#X100, Sigma-Aldrich) in 1× PBS]} was added and incubated for 2 h at room temperature. BBT was then removed and the plates were washed twice with PBT for 5 min each. The samples were then incubated with the polyclonal primary antibody anti-Dilp2 (*Drosophila* insulin-like peptide; 1:2000 in BBT) raised in rabbit against amino acids 108–118 (TRQRQGIVERC) of the Dilp2 protein (Eurogentec, Rue du Bois Saint-Jean, Belgium) for 2 h at room temperature with constant shaking. The wells were washed 3 times with PBT for 5 min each. Anti-Rabbit IgG HRP-linked secondary antibody (#7074, Cell Signaling Technology) was added to the wells and incubated for 1 h with constant shaking. The secondary antibody solution was removed and the wells were washed 3 times with PBT with each wash lasting 5 min. TMB (3,3′,5,5′-tetramethylbenzidine) substrate solution was added to the wells and incubated for 10 min. Finally, 1 mol l^−1^ H_2_SO_4_ was added to halt the reaction. Absorbance was measured at 450 nm using the TECAN Infinite M200 Pro Multimode plate reader. The readings were noted and the data were plotted using GraphPad Prism.

### Immunohistochemistry

The experimental protocol for training was followed and the brains of day 13 experimental and control flies were dissected in ice-cold PBS. The tissue was fixed in 4% paraformaldehyde (#P6148, Sigma-Aldrich) for 30 min and washed 3 times with PBT. Blocking was carried out by adding BBT followed by incubation for 45 min at room temperature. Tissues were incubated with anti-Dilp2 polyclonal primary antibody (1:500 in BBT) overnight at 4°C. After PBT washes Alexa Fluor^®^ 488 Goat Anti-Rabbit IgG secondary antibody (#A27034, Invitrogen) (1:500 in BBT) was added and incubated for 2 h at room temperature. Post-incubation, the tissues were given thorough washes with PBT before adding the mounting medium. The brains were mounted on a slide and imaged using a Zeiss LSM 880 microscope. The images were analysed using ImageJ and the corrected total cell fluorescence (CTCF) value was calculated. The data were plotted and analysed with GraphPad Prism.

### Whole-body transcriptomics

Post-training, day 13 fed and trained flies (15 per replicate) were flash-frozen at 0 and 28 h of starvation. Three replicates for the *t*_0_ time point (prior to the induction of starvation) and 2 replicates for the *t*_L_ time point (after 28 h of starvation) were taken. The samples were subsequently used to extract high-quality RNA and carry out RNA sequencing using the Illumina Novaseq NGS platform (via miBiome, Maharashtra, India). The standard sequencing parameters were 2×150 paired-end reads to generate a total of 20 million paired-end reads corresponding to 3 GB data per sample.

Differential gene expression was analysed using the DESeq function from the DESeq2 package with a false discovery rate (FDR) cut-off of <0.05 and a minimum expression log_2_-fold change (FC) of ≥2/1. The significant differentially expressed genes were taken and run on shinygo v0.80: Gene Ontology Enrichment Analysis server (http://bioinformatics.sdstate.edu/go/) with *Drosophila melanogaster* as the reference.

### Statistical analyses

All the analyses and figure preparations were performed using GraphPad Prism version 10.6.1 (GraphPad, Boston, MA, USA) unless specified otherwise. The transcriptomics dataset was analysed by miBiome using the standard bioinformatics pipeline. All the experiments were performed in biological replicate with specific ‘*n*’ numbers denoted in the figure legends.

The survival curves for starvation, oxidative and desiccation stresses were analysed by the Mantel–Cox test (log-rank) using Online Application for Survival Analysis 2 (OASIS 2, Pohang, South Korea). For the rest of the data, depending on the dataset and distribution, the following tests were employed: two-way ANOVA with Tukey's multiple comparison test, two-tailed Student's *t*-test with Welch's correction and Mann–Whitney *U*-test. The specific statistical tests used for individual datasets are mentioned in the figure legends. All analyses were performed keeping the significance threshold of *P*<0.05 and data are presented as means±s.e.m.

### AI use

AI (Grammarly, Google Docs and ChatGPT) was used for correction of spelling and grammar and for refining parts of the text. The authors subsequently reviewed and edited the content as necessary and take full responsibility for the publication's final content.

## RESULTS

### Exposure to brief bouts of starvation enhances starvation resistance in *Drosophila melanogaster*

To investigate the impact of prior exposure to starvation on the ability of *D. melanogaster* to endure prolonged starvation, we developed a robust training protocol involving daily bouts of food deprivation. Flies were provided with food for 6 h daily, beginning at 10:00 h, followed by an 18 h starvation period beginning at 16:00 h (Fig. 1A). Under standard conditions, flies were continuously provided with food in vials, with no restrictions in terms of access to food. The feeding schedule in our protocol aligns with the observed peaks in feeding behaviour of *Drosophila* (Xu et al., 2008). To mitigate excessive mortality and minimize stress, a recovery day was included every fourth day. This training regimen continued for 12 days, with final measurements taken on day 13. Flies subjected to this protocol, referred to as ‘trained’ flies, were compared with a control group of ‘fed’ flies that were normally fed for the same duration.

Our goal was to examine the hormetic response to food deprivation stress. To this end, we exposed both trained and fed flies to prolonged starvation and monitored mortality over time. Remarkably, the trained flies exhibited significantly higher tolerance to starvation compared with the fed control group, a phenomenon we term enhanced starvation resistance (ESR) (Fig. 1Bi). The median mortality plot revealed a notable increase in survival among trained flies under starvation conditions compared with their fed counterparts (Fig. 1Bii). These findings demonstrate that our protocol effectively induces a hormetic response, enhancing resistance to starvation stress in *D. melanogaster*.

### Trained flies exhibit modulations in energy expenditure during chronic starvation

**Fig. 2.**
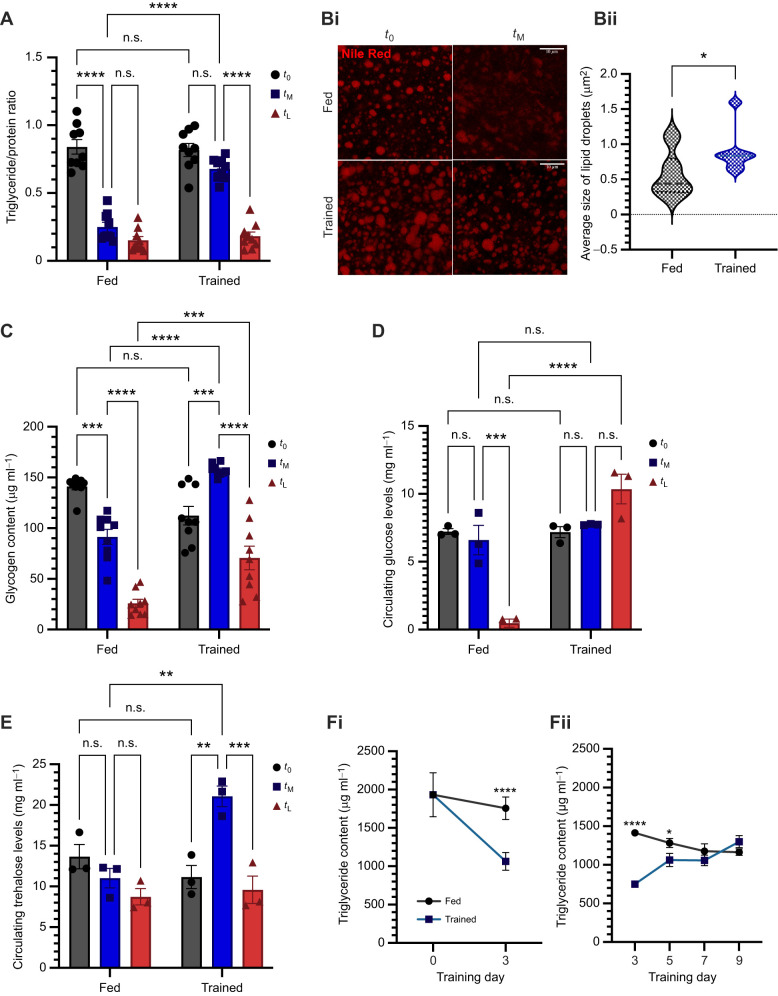
**Trained flies show alteration in starvation-induced depletion of energy reserves.** (A) Triglyceride levels of fed and trained flies at pre-starvation (*t*_0_), mid-starvation (*t*_M_) and late starvation (*t*_L_) (*n*=9 per time point). (Bi) Nile Red staining of fat body dissected from fed and trained flies at *t*_0_ and *t*_M_. (Bii) Quantification of average lipid droplet size in the fat body tissue at *t*_0_. (C) Glycogen content of the flies at *t*_0_, *t*_M_ and *t*_L_ (*n*=9 per time point). (D) Haemolymph glucose levels of the flies at *t*_0_, *t*_M_ and *t*_L_ (*n*=3 per time point). (E) Circulating trehalose levels of the flies at *t*_0_, *t*_M_ and *t*_L_ (*n*=3 per time point). (Fi) Triglyceride content of fed and trained flies before initiation of training at day 0 and at day 3 of training (day 0: *n*=8, day 3 fed: *n*=9, day 3 trained: *n*=8). (Fii) Triglyceride levels of fed and trained flies on days 3, 5, 7 and 9 of training (*n*=3). A, Bi, C, D, E and Fii were analysed with two-way ANOVA with Tukey's HSD *post hoc* test for multiple comparisons, Bii was analysed with a Mann–Whitney test, Fi was analysed using Student's two-tailed *t*-test with Welch's correction (*P<0.05, **P<0.01, ***P<0.001, ****P<0.0001). Data are presented as means±s.e.m*.*

To explore the mechanisms underlying ESR in trained flies, we investigated their energy reserves. Initial assays of triglycerides, glycogen, glucose and trehalose immediately post-training revealed no significant differences between trained and fed flies (Fig. 2A,C–E). This finding led us to hypothesize that the observed differences may lie in the utilization of energy reserves during starvation. To test this, we assayed energy reserves at three time points: *t*_0_ (prior to induction of starvation), *t*_M_ (mid-starvation, after 16 h of starvation) and *t*_L_ (late starvation, after 28 h of starvation). Triglycerides, a primary energy source during nutrient deprivation (Grönke et al., 2007), exhibited a characteristic decline in fed flies during starvation. While trained flies displayed a similar trend, they maintained higher triglyceride levels at the mid-starvation point compared with fed controls, suggesting an altered pattern of utilization of lipid stores (Fig. 2A). To further investigate this, we analysed lipid droplet morphology within the fat body, a key metabolic tissue. Post-training, the fat body was stained with Nile Red at the *t*_0_ and *t*_M_ time points, which revealed that trained flies possessed larger lipid droplets than their fed counterparts (Fig. 2Bi,ii), which was surprising as the triglyceride levels were not significantly different between fed and trained flies. However, upon exposing the flies to 16 h of starvation, the lipid utilization was found to be reduced in the trained fat tissue (Fig. 2Bi). Glycogen, a crucial carbohydrate reserve, also plays a vital role in starvation survival (Yamada et al., 2018). In fed flies, glycogen levels decreased progressively during starvation (Fig. 2C). Interestingly, trained flies, despite starting with slightly lower basal glycogen levels, maintained higher glycogen levels during mid- and late stages of starvation. Notably, trained flies exhibited a temporary increase in glycogen levels at *t*_M_ compared with pre-starvation levels (*t*_0_), indicating a potential modulation in the balance of glycogen synthesis and breakdown, which probably contributes to delayed mortality.

To assess the utilization of circulating energy reserves, we measured glucose and trehalose levels in the haemolymph under starvation conditions. As starvation progressed, glucose levels in fed flies declined (Fig. 2D). In contrast, trained flies maintained glucose levels comparable to their baseline (*t*_0_), even after 28 h of starvation (*t*_L_). Similarly, trehalose levels in fed flies decreased slightly, albeit not significantly so (Fig. 2E). Although initial trehalose levels were comparable between fed and trained groups, trained flies exhibited a marked increase in circulating trehalose at *t*_M_, which subsequently declined by *t*_L_.

These findings suggest that trained flies undergo significant metabolic rewiring, allowing them to optimize energy reserve utilization and maintain significantly higher levels of energy reserves during the early phases of starvation. These results suggest that the metabolic adaptation aids trained flies to endure starvation stress effectively, culminating in prolonged survival and ESR.

### Early training impacts triglyceride but not glycogen levels

The experiments above primarily focused on the effects of training on energy reserves post-training. A remaining question was how these reserves are modulated during the training period itself. To address this, we measured the levels of triglycerides and glycogen at regular intervals – on days 0, 3, 5, 7 and 9 of training. To begin with, the triglyceride levels in the two sets of flies were the same (Fig. 2Fi). Interestingly, triglyceride levels in trained flies were reduced on day 3 and day 5, but recovered to levels comparable to those of fed flies by day 7 (Fig. 2Fi,ii). This indicated a direct consequence of prolonged starvation during early training, and an effect of training on starvation resilience on later days (Fig. 2Fi,ii). In contrast, glycogen levels did not vary significantly throughout the training period in flies that were undergoing training and did not show any major deviation from those of the fed counterparts (Fig. S1B). These results underscore the distinct metabolic responses elicited during the training regimen.

### Trained flies exhibit reduced total activity and enhanced cross-tolerance without altered feeding behaviour

**Fig. 3.**
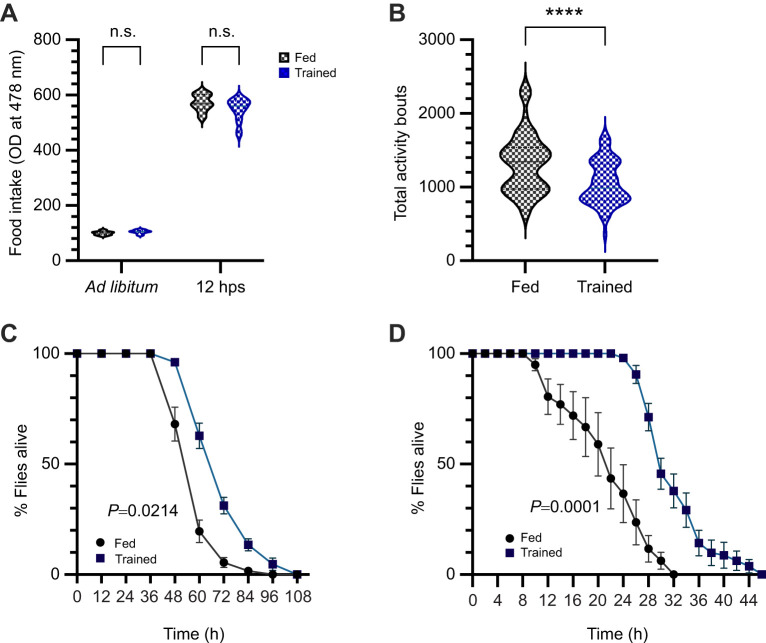
**Training impacts behaviour and stress responses.** (A) Quantification of *ad libitum* (*n*=8) and 12 h post-starvation (hps; *n*=12) feeding of the trained flies in comparison to the fed flies*.* (B) The 5 day cumulative activity rest rhythm profile of the trained and fed flies (*n*=64). (C) Survival under oxidation stress (fed: *n*=9, trained: *n*=8). (D) Survival under desiccation stress (fed: *n*=8, trained: *n*=9). A was analysed using two-way ANOVA with Tukey's HSD *post hoc* test for multiple comparisons; B was analysed using a non-parametric Mann–Whitney test; C and D were analysed using a log-rank test (*P<0.05, **P<0.01, ***P<0.001, ****P<0.0001). Data are presented as means±s.e.m*.*

To investigate behavioural adaptations induced by training, we examined feeding and activity patterns in both trained and fed flies. We hypothesized that early starvation exposure might influence baseline feeding behaviour if the flies had experienced nutrient deprivation. Additionally, we considered that an enhanced hunger-driven feeding response could be a key mechanism for improving resilience against chronic starvation in trained flies. To test these possibilities, we measured food intake using a colorimetric assay with a food dye both under *ad libitum* conditions and after 12 h of starvation. Our results showed that fed and trained flies exhibited similar feeding responses to starvation; there were no significant differences in basal or starvation-induced feeding between the two groups (Fig. 3A). This suggests that training does not directly affect feeding behaviour.

Energy conservation could be achieved through behavioural modifications, such as altered activity levels. To explore this, we examined the activity–rest rhythms of the flies post-training. Over 5 days of monitoring, trained flies exhibited significantly lower total activity compared with fed flies (Fig. 3B). This reduction in activity implies potential energy conservation in response to the training regimen.

A critical feature of hormesis is its ability to confer tolerance to a variety of stressors beyond the primary one. To test whether ESR extends to other stressors, we subjected the flies to oxidative stress after training. Trained flies demonstrated significantly greater survival under oxidative stress compared with fed flies (Fig. 3C). Furthermore, we evaluated their resistance to desiccation, finding that trained flies exhibited heightened desiccation resistance (Fig. 3D). These findings suggest that the training regimen imparts a broad, cross-tolerance to diverse stressors, supporting the notion of a hormetic stress-adaptation mechanism.

### Transcriptomic profiling reveals altered starvation response in trained flies

**Fig. 4.**
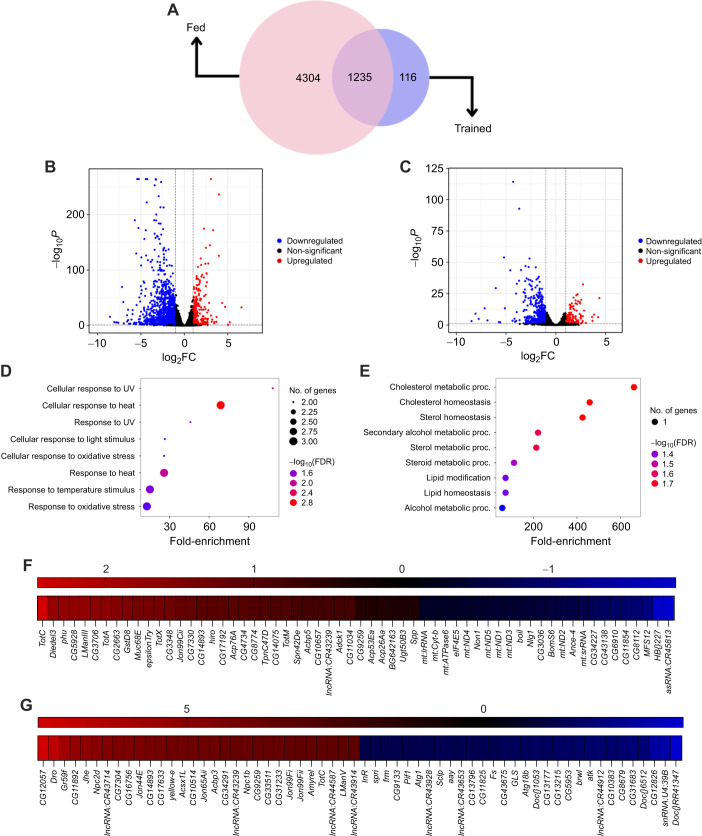
**Training alters response to starvation.** (A) Venn diagram showing the overlap of differentially expressed genes between fed and trained flies as the starvation response from *t*_0_ to *t*_L_. (B,C) Volcano plot of upregulated and downregulated genes between *t*_0_ and *t*_L_ in fed flies (B) and trained flies (C) [log_2_fold change (FC)≥1]. (D) Biological processes upregulated in trained flies at *t*_0_ relative to fed flies. (E) Biological processes downregulated in trained flies at *t*_0_ relative to fed flies. (F) Heat map of the top 25 genes upregulated and downregulated in trained flies at *t*_0_ (based on log_2_FC). (G) Heat map of the top 25 genes upregulated and downregulated in trained flies at *t*_L_ (based on log_2_FC).

To investigate the molecular reprogramming underlying the ESR, we performed RNA sequencing on fed and trained flies at two time points: pre-starvation (*t*_0_) and late starvation (*t*_L_, after 28 h of starvation). Differential gene expression analysis, using *t*_0_ as the baseline within the fed and trained groups, allowed us to identify starvation-induced changes that were common or unique to fed and trained flies. In fed flies, 5539 genes were differentially expressed (adjusted *P*<0.05) after 28 h of starvation (*t*_L_), with 3118 genes downregulated and 2421 upregulated. In contrast, trained flies exhibited a more constrained transcriptional response, with only 1351 differentially expressed genes at *t*_L_: 975 downregulated and 376 upregulated. Between the fed and trained groups, 1235 genes exhibited common starvation-induced changes, whereas 4304 genes were uniquely altered in fed flies, and only 116 genes were specifically altered in trained flies (Fig. 4A). This stark contrast is illustrated in volcano plots (log_2_FC≥±1), where fed flies display a broader range of gene expression changes (Fig. 4B), while trained flies exhibit a more selective and refined transcriptional regulation in response to starvation (Fig. 4C). These findings suggest that fed flies experience widespread transcriptional alterations in response to starvation, whereas trained flies demonstrate a restrained and targeted response.

To further explore the transcriptomic distinctions induced by training, we compared the differential gene expression of trained flies relative to that of fed flies at *t*_0_ to provide clarity regarding the genes that are basally different in the fed and trained flies due to training. As expected, trained flies exhibited upregulation of genes involved in various stress response pathways, including heat stress, oxidative stress and ultraviolet radiation (Fig. 4D). Interestingly, most of the downregulated genes at this stage were associated with lipid, cholesterol and alcohol metabolism (Fig. 4E). This pattern suggests that trained flies pre-emptively activate stress response pathways while downregulating metabolic pathways, potentially contributing to their enhanced survival capability. Gene ontology (GO) enrichment analysis identified biological processes associated with genes selectively upregulated or downregulated in fed and trained flies during starvation (Fig. S2). These results underscore key molecular differences in starvation responses between the two groups, probably underpinning the enhanced starvation resistance observed in trained flies.

A heatmap of the top 25 upregulated and downregulated genes (ranked by fold-change) in trained flies compared with fed flies at *t*_0_ revealed significant activation of genes involved in stress signalling pathways (Fig. 4F), which includes members of the Turandot family (*TotC*, *TotA* and *TotX*). Genes encoding enzymes (*LManIII*, *GstD8*, *hiro*, *Acp76A*, *εTry*) and metabolic regulators (*CG17192*, *Acbp5*) were also upregulated in trained flies. Notably, downregulated genes at this stage encompassed mitochondria-specific genes and genes critical for neuromuscular junction function, translation initiation and transmembrane transport (Fig. 4F). Upon starvation (*t*_L_), the transcriptomic profile of trained flies underwent significant changes (Fig. 4G). Many upregulated genes were linked to immune response and ecdysteroid hormone synthesis, while downregulated genes were associated with autophagy, DNA repair and several non-coding RNAs. While stress signalling pathway activation appears to be a primary mechanism underlying the enhanced starvation resistance of trained flies, the roles of other differentially expressed genes across pre-starvation and post-starvation stages require further investigation.

Interestingly, expression of a few genes regulated by insulin signalling was significantly reduced in trained flies compared with fed flies under nutrient-deprived conditions, including *inr*, the gene encoding the *Drosophila* insulin receptor and a key component of the insulin signalling pathway (Fig. 4G; Fig. S3A). Insulin signalling, a crucial nutrient-responsive pathway that regulates energy homeostasis, is typically downregulated during food deprivation. Given that *inr*, eIF4E-binding protein (*4ebp*) and the lipase Brummer (*bmm*) gene expression is negatively regulated by insulin signalling (Jünger et al., 2003; Zinke et al., 2002; Puig et al., 2003), this suggests increased insulin pathway activity in trained flies during starvation. To further investigate this observation and elucidate the role of insulin signalling under repeated nutritional stress, we validated several well-established targets within the insulin signalling pathway.

### Trained flies maintain high insulin signalling under starvation conditions

**Fig. 5.**
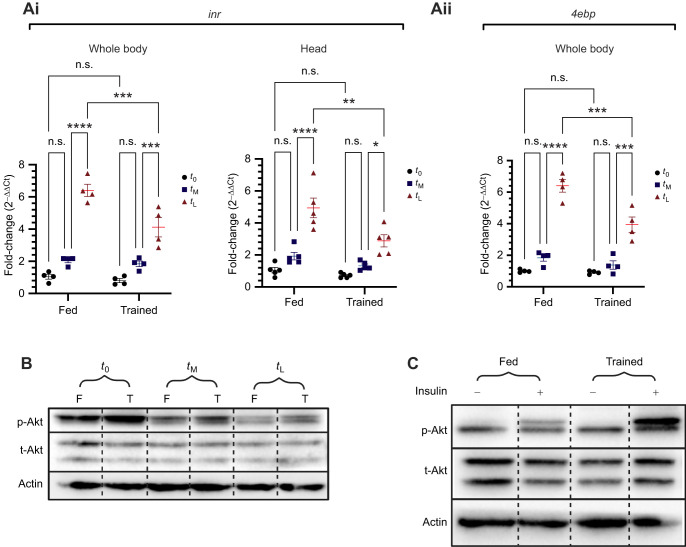
**Changes in insulin signalling in response to starvation are different in fed and trained flies.** (Ai) Transcript levels of *inr* in head (*n*=5) and whole body (*n*=4) of the fed and trained flies at *t*_0_, *t*_M_ and *t*_L_. (Aii) Transcript levels of *4ebp* of the fed and trained flies at *t*_0_, *t*_M_ and *t*_L_ (*n*=4). (B) Western blot of phosphorylated (p-Akt) and total (t-Akt) levels of fed (F) and trained (T) flies at *t*_0_, *t*_M_ and *t*_L_ (representative image). (C) Western blot of p-Akt and t-Akt levels of fed flies following incubation with 1 μmol l^−1^ insulin (representative image). Ai and Aii were analysed using two-way ANOVA with Tukey's HSD *post ho*c test for multiple comparisons (*P<0.05, **P<0.01, ***P<0.001, ****P<0.0001). Data are presented as means±s.e.m*.*

To validate these findings, we performed RT-qPCR to analyse the levels of insulin signalling target genes at three time points: pre-starvation (*t*_0_), mid-starvation (*t*_M_) and late starvation (*t*_L_). As expected, both trained and fed flies showed increased expression of *4ebp*, *bmm* and *inr* in response to starvation. However, consistent with the RNA sequencing results, trained flies exhibited significantly lower expression levels of *inr* (Fig. 5Ai), *4ebp* (Fig. 5Aii), and *bmm* (Fig. S3B) when compared with fed flies, indicating higher insulin signalling, at the *t*_L_ time point. These findings suggest that trained flies maintain higher insulin signalling despite prolonged starvation. To further corroborate these results, we examined the phosphorylation status of Akt, a key upstream effector molecule in the insulin signalling pathway. Activation of the pathway leads to phosphorylation of Akt. Western blot analysis of phosphorylated Akt (p-Akt) levels at *t*_0_, *t*_M_ and *t*_L_ revealed a reduction of p-Akt levels in both fed and trained flies in response to starvation. However, elevated p-Akt levels in trained flies were observed in comparison to fed flies, particularly at the mid-point and late-point of starvation (Fig. 5B; Fig. S3C). These results that measure insulin signalling activity confirm that trained flies maintain higher insulin signalling even in response to longer periods of starvation.

In addition to downstream signalling, we explored potential changes occurring at the level of the ligand. In adult *Drosophila*, energy homeostasis is regulated by three major insulin-like peptides (DILPs) – DILP2, DILP3 and DILP5 – secreted by seven pairs of neurosecretory cells known as insulin-producing cells (IPCs). The release of DILP2 and transcriptional regulation of *dilp3* and *dilp5* are dependent on nutritional status (Ikeya et al., 2002; Broughton et al., 2005). To identify potential changes, we analysed transcript levels of *dilp2*, *dilp3* and *dilp5* in head mRNA, as well as DILP2 protein levels within the IPCs and haemolymph (Fig. S4). Notably, only *dilp5* mRNA levels were reduced in response to starvation in fed flies (Fig. S4A″). While *dilp2* and *dilp3* mRNA levels remained unchanged during mid-starvation (Fig. S4A,A′), at the late starvation time point, *dilp3* levels exhibited an increase (Fig. S4A′). However, when comparing *dilp* mRNA levels between fed and trained flies during starvation, no significant differences were observed, except for *dilp3*, which did not show any changes in trained flies at the late starvation time point (Fig. S4A,A′,A″). Circulating levels of DILP2 showed a slight, non-significant reduction in fed flies during starvation, while in trained flies, DILP2 protein levels in the haemolymph remained unaffected by starvation (Fig. S4B). The levels of DILP2 protein in the IPCs also did not differ between the fed and the trained flies (Fig. S4C). These findings confirm that trained flies maintain robust insulin signalling during starvation, probably as a direct consequence of the training protocol, without notable changes in ligand levels. The sustained insulin signalling may contribute to the observed enhanced starvation resistance in trained flies.

As an extension of the above findings, we wondered whether the feeding–starvation regime imparted the trained flies with a higher degree of insulin sensitivity. To test this, we exposed the dissected fat body tissue of the day 13 fed and trained flies to 1 µmol l^−1^ of human insulin and measured p-Akt levels by western blot. Interestingly, fatbody tissue from trained flies subjected to insulin exposure mounted a higher insulin signalling response, evidenced by higher p-Akt levels in comparison to the tissue from fed flies, which displayed lower p-Akt levels and hence lower insulin signalling (Fig. 5C; Fig. S4D). Thus, trained flies exhibit higher insulin sensitivity in comparison to fed flies, which could be the reason why they can sustain higher insulin signalling during starvation and survive better.

### Insulin signalling is essential for the establishment of enhanced starvation resistance

**Fig. 6.**
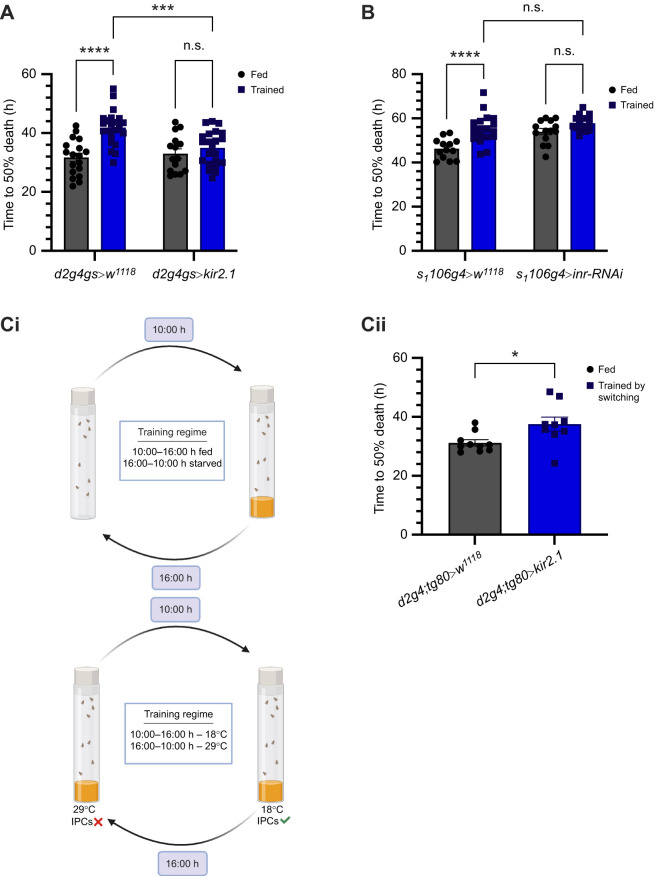
**Blocking insulin signalling hinders establishment of enhanced starvation resistance (ESR).** (A) Inactivating insulin-producing cells by hyperpolarizing neurons inhibits ESR (*d2g4gs>w^1118^* fed: *n*=18, *d2g4gs>w^1118^* trained: *n*=24, *d2g4gs*>*kir2.1* fed: *n*=15, *d2g4gs>kir2.1* trained: *n*=22). (B) Blocking peripheral insulin signalling by downregulating *inr* in the fat body leads to loss of ESR (*s_1_106g4*>*w^1118^* fed: *n*=13, *s_1_106g4>w^1118^* trained: *n*=19, *s_1_106g4>inr-RNAi* fed: *n*=14, *s_1_106g4>inr-RNAi* trained: *n*=17). (Ci) Protocol for inducing pulses in insulin signalling during the training regime. Created in BioRender. (https://BioRender.com/e27a192) (Cii) Median survivorship of flies trained by switching shows pulses of insulin signalling can induce ESR (*d2g4;tg80>w^1118^*: *n*=10, *d2g4;tg80>kir2.1*: *n*=9). A and B were analysed with two-way ANOVA with Tukey's HSD *post hoc* test for multiple comparisons, Ci was analysed using a Mann–Whitney test (*P<0.05, **P<0.01, ***P<0.001, ****P<0.0001). Data are presented as means±s.e.m*.*

Having established that insulin signalling is maintained at higher levels under starvation in trained flies, we sought to understand how alterations in this pathway during training influence the establishment of the ESR phenotype. To investigate this, we first disrupted IPC-derived systemic insulin signalling during the training phase by expressing the inward rectifying potassium ion channel *Kir2.1* specifically in IPCs using the *dilp2-GAL4-geneswitch* (*dilp2-GS*) driver. This approach limited inactivation of the IPCs to the training period. When subjected to chronic starvation, trained flies with reduced IPC activity failed to exhibit ESR (Fig. 6A). In contrast, trained flies with normal IPC activity showed the expected increased starvation tolerance. Moreover, in trained flies, inactivation of IPCs led to a partial loss of the triglyceride utilization pattern observed in trained flies that had normal IPC activity (Fig. S5A,B). This suggests that IPC activity during training has a crucial influence on triglyceride utilization during starvation and enhanced resistance to starvation.

We next explored the role of peripheral insulin signalling by targeting the fatbody, a metabolically active tissue critical for energy homeostasis. Using the GeneSwitch system, we expressed *UAS-inr-RNAi* in the fat body with the *S_1_106-GAL4-geneswitch* driver (*S_1_106-GS*) during the training period. Downregulation of insulin receptor expression in the fatbody led to starvation resistance in fed flies, and the training protocol did not further enhance the resistance of flies to starvation (Fig. 6B). Therefore, similar to perturbation of IPC function, peripheral downregulation of insulin signalling abolished the ESR phenotype in trained flies (Fig. 6B), while control flies retained the training effect and displayed increased survival under starvation. However, downregulation of fatbody insulin signalling in trained flies led to a triglyceride utilization pattern similar to that of their counterparts with normal fatbody insulin signalling (Fig. S5C,D). This suggests that the effect of training was lost on flies with impaired peripheral insulin signalling, despite these flies maintaining the capacity for controlled utilization of triglyceride levels. These results underscore the critical role of both IPC-derived systemic and peripheral insulin signalling during training in establishing the hormetic ESR phenotype, the key differences observed in the utilization of energy stores and the differential contribution of this to ESR.

### Cycling of insulin signalling is critical for enhanced starvation resistance

Under normal conditions, insulin signalling dynamically responds to fluctuations in nutrient availability, activated during feeding and diminished during starvation (Ikeya et al., 2002; Wang et al., 2008; Bisen et al., 2025). Our training protocol involved alternating cycles of feeding and starvation, which leads to fluctuations in insulin signalling (Fig. S6), probably inducing multiple oscillations in insulin signalling during the training period. Given that complete inactivation of insulin signalling disrupts ESR, we hypothesized that the cycling of insulin signalling during training is critical for establishing ESR.

To test this hypothesis, we employed a temperature-sensitive *dilp2-GAL4;tubulin-GAL80^ts^* line crossed with *UAS-Kir2.1*, enabling temporal inhibition of IPC activity. Normally fed flies were maintained at the restrictive temperature (18°C) for 6 h from 10:00 h to 16:00 h – the feeding period in the training protocol – to allow normal IPC activity. Subsequently, these flies were shifted to the permissive temperature (29°C) for 18 h from 16:00 h, corresponding to the starvation period of the training protocol. This temperature shift allowed normal IPC activity for 6 h and induced IPC inactivation for 18 h, simulating a regime similar to the training protocol (Fig. 6Ci). We confirmed the effectiveness of our protocol by assessing DILP2 levels within the IPCs at two time points: immediately before switching to 18°C at 10:00 h and immediately before switching to 29°C at 16:00 h (Fig. S7A–B′). Remarkably, genetically trained flies subjected to this protocol exhibited enhanced starvation tolerance, similar to the behaviour observed under standard training conditions (Fig. 6Cii; Fig. S7C). These results demonstrate that oscillatory activation and inactivation of IPCs, replicating the training protocol, is sufficient to induce the starvation-resistant enhanced stress response. These findings highlight the importance of insulin signalling oscillations during our training protocol in establishing the ESR phenotype, offering new insights into the mechanistic basis of metabolic adaptation in response to repeated starvation cycles. Thus, our results suggest that the activity of the insulin signalling pathway during the training process and maintenance of higher insulin signalling in response to chronic exposure to starvation contributes to the enhanced resistance phenotype. This study also highlights the role of insulin signalling oscillations in developing ESR.

## DISCUSSION

In this study, we investigated the metabolic and molecular mechanisms underlying hormetic responses in *D. melanogaster* by exposing flies to mild nutritional stress, which enhances resilience to prolonged nutrient deprivation.

Previous research in *Drosophila* has demonstrated that early-life stressors – including nutritional, oxidative, hypoxic and thermal stress – have lasting effects on adult metabolism and physiology, potentially increasing tolerance to chronic stress in later life. For example, poor larval nutrition reduces fecundity and alters metabolic states, contributing to enhanced adult nutrient stress tolerance (Klepsatel et al., 2020; Kolss et al., 2009; Rehman and Varghese, 2021). Similarly, mild early-life heat stress induces mitochondrial and physiological changes that improve stress tolerance and longevity (Hunter-Manseau et al., 2025). However, not all early-life stress exposures lead to increased adult resilience, and the tolerance mechanisms elicited do not always involve cellular adaptations. Developmental exposure to hypoxia, for instance, reduces adult starvation resistance and shortens lifespan, without conferring hypoxia tolerance (Polan et al., 2020). Likewise, early oxidative stress induced by low-dose reactive oxygen species (ROS) exposure extends lifespan through microbiome remodelling rather than direct cellular adaptation (Obata et al., 2018). These findings underscore the profound and lasting impact of early environmental stress on metabolic health and lifespan. Our study further demonstrates that prior exposure to mild stress induces adaptive responses that enhance survival under chronic stress conditions in adult *Drosophila*.

The storage and utilization of nutrient reserves are critical for meeting metabolic demands during food deprivation, thereby influencing survival outcomes (Heier and Kühnlein, 2018; Grönke et al., 2007; Palanker et al., 2009; Rehman and Varghese, 2021). Previous studies have shown that flies reared on a low-protein diet exhibit increased starvation survival due to elevated triglyceride reserves and enhanced lipid mobilization (Katewa et al., 2012). Although brief starvation periods improved starvation resistance in our experiments, they did not alter basal metabolic states. However, trained flies exhibited distinct shifts in the utilization patterns of both tissue and circulating energy stores (Fig. 2A–F). In normally fed flies, energy reserves declined progressively during the mid-to-late phases of starvation (Fig. 2A–E). In contrast, trained flies maintained their energy reserves through the mid-starvation phase, with glycogen and trehalose levels even increasing. Our transcriptomic data indicate a starvation-induced increase in transcript levels in the trained flies in comparison to the fed flies of *tps1* (*trehaslose-6-phosphate synthase 1*), *gbs-70e* (*glycogen binding subunit 70E*), *ugp* (*UDP-glucose pyrophosphorylase*) and *pgm1* (*phosphoglucose mutase 1*), which are genes coding for enzymes that could lead to the accumulation of trehalose and glycogen (Chen et al., 2002; Yoshida et al., 2016; Kerekes et al., 2014; Elbein et al., 2003; Verrelli and Eanes, 2001), thereby contributing to enhanced starvation resilience in the trained flies (Table S2). At the late starvation phase, depletion of glycogen and trehalose was minimal, and glucose levels remained higher than at the onset of starvation. Notably, only triglyceride levels showed significant depletion over 28 h of starvation (Fig. 2A). These findings suggest that differential energy reserve utilization contributes to enhanced starvation resistance. The delayed utilization of triglycerides may have contributed to altered synthesis and usage of other energy reserves despite ongoing starvation. Additionally, trained flies exhibited larger lipid droplets in their fat bodies compared with their non-trained counterparts. After 16 h of starvation, lipid depletion in these droplets was less pronounced (Fig. 2B). The expression of the lipase gene *bmm* was maintained at lower levels in trained flies during starvation (Fig. S3B), possibly causing the effects on triglyceride utilization. Similar patterns were observed in adult flies subjected to poor early-life stage nutrition and in *bmm* mutants (Rehman and Varghese, 2021; Grönke et al., 2005), demonstrating that regulated lipid store utilization enhances survival. These findings suggest that prior exposure to nutritional stress fine-tunes energy expenditure, promoting survival under prolonged food deprivation.

Feeding behaviour is closely linked to nutritional availability and metabolic status (reviewed in Mahishi et al., 2024). Notably, our study found no significant differences in *ad libitum* or starvation-induced food intake between fed and trained flies (Fig. 3A), similar to the metabolic states, ruling out nutrient deprivation. Dietary status has been reported to affect locomotion in flies and is considered to be an adaptation to promote foraging for food in response to nutrient deprivation (Katewa et al., 2012; Krittika and Yadav, 2020; Bross et al., 2005). However, the trained flies in our study exhibited lower overall activity compared with their fed counterparts, suggesting an energy conservation mechanism that helps preserve metabolic reserves (Fig. 3B). This highlights a potential adaptive strategy worthy of further exploration. Earlier reports suggest that energy conservation in *Drosophila* during low-nutrient conditions is achieved by reducing locomotor activity. Flies selected for increased starvation resistance exhibit decreased movement, indicating an energy-saving adaptation (Schwasinger-Schmidt et al., 2012). Additionally, diminished AMP-activated protein kinase (AMPK) signalling results in lower baseline locomotor activity, which is considered an energy-saving mechanism (Johnson et al., 2010). Thus, reduced activity in trained flies could be an adaptive response to conserve energy, thereby aiding survival during extended starvation, a better strategy than altering the basal metabolic state or *ad libitum* feeding. As expected, trained flies displayed enhanced resistance to oxidative and desiccation stress, suggesting cross-tolerance to multiple stressors, aligning with hormetic adaptation (Fig. 3C,D). These findings emphasize the physiological and behavioural benefits of hormesis.

While fed flies experienced large-scale transcriptional changes in response to starvation, trained flies showed a restrained response (Fig. 4). Our transcriptomic analysis identified gene expression changes linked to stress response pathways and insulin signalling in trained flies, highlighting its role in enhancing starvation resilience (Fig. 4; Fig. S3). *Drosophila* insulin-like peptides (DILPs) are a family of ligands that regulate growth, metabolism, reproduction and lifespan. The *Drosophila* genome encodes eight DILPs (DILP1–8), each with distinct expression patterns and physiological functions (Brogiolo et al., 2001; Grönke et al., 2010; Cao and Brown, 2001; Colombani et al., 2012; Slaidina et al., 2009). Among them, DILP2, DILP3 and DILP5 are expressed in insulin-producing cells of the brain, where they regulate metabolism, growth and development (Ikeya et al., 2002; Rulifson et al., 2002; Nässel et al., 2013). As central regulators, DILPs integrate environmental cues with internal physiological states to maintain organismal homeostasis (Ikeya et al., 2002; Hong et al., 2012; Varghese et al., 2010; Slaidina et al., 2009; Bai et al., 2012; Söderberg et al., 2012). Despite their crucial role, expression levels of *dilp2*, *dilp3* and *dilp5* mRNA, as well as DILP2 protein levels, remained largely unchanged in trained flies (Fig. S4A–C). While no significant changes were observed in ligand expression, downstream insulin signalling responded markedly to starvation. In *Drosophila*, the insulin signalling pathway modulates the expression of key target genes such as *4ebp* (encoding eIF4E binding protein), *inr* (insulin receptor) and *bmm* (Brummer, an ATGL-like lipase) to regulate growth and metabolism in response to environmental and physiological cues (Bai et al., 2012; Puig and Tjian, 2005; Puig et al., 2003; Wang et al., 2011). Under reduced insulin signalling, the transcription factor dFOXO translocates to the nucleus, activating *4ebp*, *inr* and *bmm* expression (Alvarez et al., 2001; Puig and Tjian, 2005; Wang et al., 2011). Another key component of insulin signalling is protein kinase B, also known as Akt, which becomes activated through phosphorylation upon pathway stimulation. Activated Akt phosphorylates downstream targets to regulate protein synthesis, glucose metabolism and cell survival (Verdu et al., 1999; Scanga et al., 2000). Typically, insulin signalling decreases during food deprivation (Ikeya et al., 2002; Sudhakar et al., 2020). In our experiment, fed flies exhibited a significant reduction in pathway activity (Fig. 5A,B; Fig. S4), while trained flies maintained higher insulin signalling even during prolonged starvation, as evidenced by lower transcript levels of *4ebp*, *inr* and *bmm*, along with elevated p-Akt levels (Fig. 5A,B; Fig. S3). These findings suggest that prior exposure to mild starvation stress and possible cyclic inactivation of insulin signalling (Fig. S6) rewires insulin responses, a critical energy regulatory mechanism, during the training phase, thereby enhancing resilience to prolonged starvation. Maintenance of insulin signalling during starvation may aid the trained flies in regulating the shift to catabolic responses. This could enable the flies to utilize their stored energy reserves in a controlled manner, thus helping them to survive longer in response to starvation. In support of this, we noticed that the expression of a crucial catabolic gene, *bmm*, is maintained at lower levels in trained flies during starvation (Fig. S3B). Moreover, we found that trained flies show enhanced sensitivity to insulin signalling (Fig. 5C), which might be the reason why they can sustain higher insulin signalling during starvation and resist the effects of starvation. Past research has reported metabolic outcomes associated with time-restricted feeding across species. In humans, early day TRF (eTRF) improves glucose levels and alters lipid metabolism in overweight individuals (Jamshed et al., 2019). eTRF has also been reported to enhance insulin sensitivity in both healthy individuals and those with metabolic disorders such as type 2 diabetes and prediabetes (Xie et al., 2022; Che et al., 2021; Sutton et al., 2018). Studies in rodent models and *Drosophila* have further highlighted the changes in insulin signalling associated with feeding–fasting cycles. In mice, TRF improves glucose tolerance and insulin sensitivity and reduces the plasma concentration of insulin (Yan et al., 2024; Chung et al., 2016). Similarly, in flies, TRF ameliorates high-fat diet-related metabolic dysfunction by promoting insulin sensitivity and decreasing glucose levels, further supporting the conserved role of insulin signalling in TRF regimes (Salgado-Canales et al., 2023).

Furthermore, we demonstrate that insulin signalling plays a key role in establishing the enhanced starvation resistance phenotype rather than being a mere consequence of training. As noted earlier, insulin signalling is tightly linked to nutritional status, increasing with food availability and decreasing under nutrient scarcity (Lee et al., 2008; Hong et al., 2012; Britton et al., 2002; Ikeya et al., 2002; Moskalev et al., 2015). To investigate the role of insulin signalling in the ESR phenotype, we reduced IPC activity and limited *inr* expression in peripheral fat tissue – methods previously used to suppress insulin signalling in *Drosophila* (Géminard et al., 2009; Sudhakar et al., 2020). As a result, trained flies no longer exhibited enhanced starvation survival (Fig. 6A,B). The effects of training on ESR are mediated by IPCs partly through the effective utilization of triglyceride stores (Fig. S5A,B). However, the mechanism is different for fatbody insulin signalling, which is also crucial for ESR but does not occur through changes in triglyceride levels (Fig. S5C,D). Our training protocol involved cyclic feeding and fasting, probably inducing pulsatile activation of insulin signalling (Fig. S6). We hypothesize that these oscillations contribute to enhanced starvation resistance. Our data on the effects of suppressing IPC activity and downregulating insulin receptor levels in peripheral fat tissue on the starvation resilience of trained flies suggest this possibility. Additionally, using a genetic switch to regulate IPC activity – allowing normal neuronal function for 6 h followed by 18 h of inactivation over 12 days (including rest days) without restricting food supply (Fig. 6Cii) – we found that cyclic IPC activity and insulin signalling are central to metabolic adaptation under starvation stress in trained flies.

In conclusion, our study demonstrates that prior exposure to mild nutritional stress induces metabolic and molecular adaptations in *D. melanogaster*, enhancing their resistance to prolonged starvation. Trained flies exhibited altered energy utilization, conserving glycogen and trehalose while primarily relying on triglyceride metabolism. These metabolic shifts coincide with reduced locomotor activity, suggesting an energy conservation strategy. Additionally, trained flies showed cross-tolerance to oxidative and desiccation stress, highlighting the broader physiological benefits of hormetic adaptation. At the molecular level, we identified insulin signalling as a key regulator of starvation resilience. While ligand expression remained unchanged, trained flies maintained higher downstream insulin signalling, probably through cyclic activation during feeding–fasting cycles. Genetic disruption to insulin signalling during training abolished starvation resistance, underscoring its functional significance. However, other signalling mechanisms may also contribute to stress resilience, and the molecular link between insulin signalling and the metabolic responses underlying enhanced starvation resistance remains unclear. Further research is needed to address these gaps.

Our findings provide new insights into how prior nutritional stress reprogrammes metabolic and hormonal pathways, paving the way for further exploration of the role of insulin signalling in hormesis.

## Supplementary Material

10.1242/jexbio.250507_sup1 Supplementary information
