# Supplementary material for: Moderate nutritional stress reprogrammes insulin responses to drive enhanced starvation tolerance in Drosophila melanogaster
Source: J Exp Biol. 2026 Jan 26;229(2):jeb250507. doi: 10.1242/jeb.250507 (PMC12891944; doi:10.1242/jeb.250507)
Supplement: Supplementary information [file jexbio-229-250507-s1.pdf]

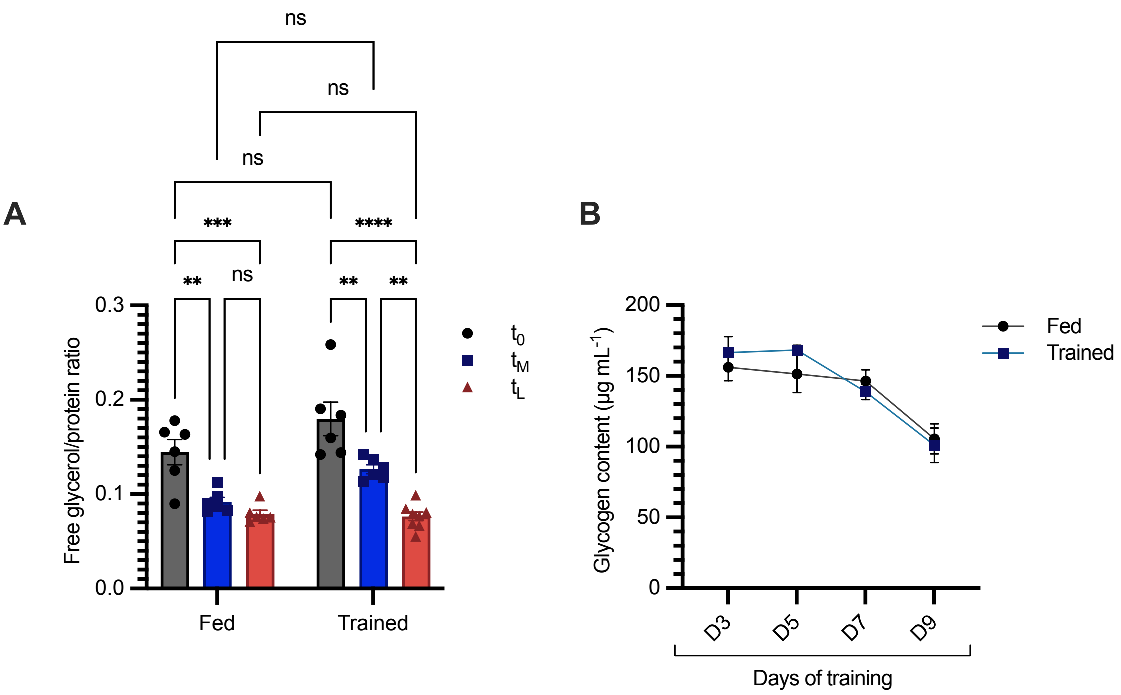

Fig. S1. Free glycerol and day-wise glycogen levels of fed and trained flies are not different A. Free glycerol levels in the fed and trained flies at t_0, t_M, and t_L*(n = 6)* B. Glycogen levels of the fed and trained flies sampled at days 3, 5, 7, and 9 of training *(n = 3 per day) [A and B, were analyzed with 2-way ANOVA with Tukey's HSD post-hoc test for multiple comparisons. p-value *<0.05; ** <0.01,*** <0.001, **** <0.0001. Data is presented as mean ± SEM]*

Journal of Experimental Biology • Supplementary information

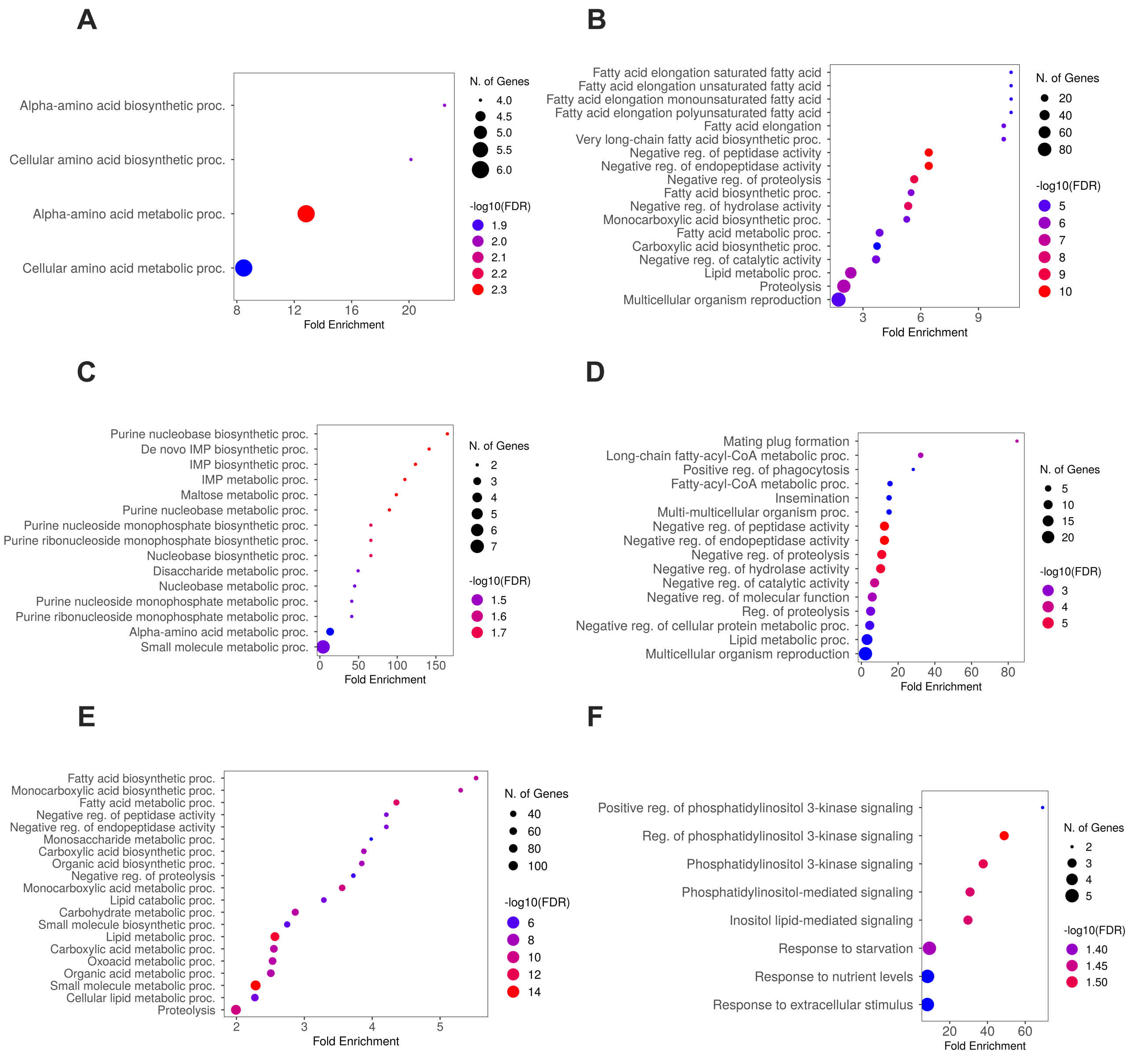

Fig. S2. Exposure to brief starvation modulates gene expression in trained flies A. Biological processes upregulated in t_0 versus t_L condition (during starvation) in fed flies *($log_2FC \geq 1$)* B. Biological processes downregulated in t_0 versus t_L condition (during starvation) in fed flies. C. Biological processes upregulated in t_0 versus t_L condition in trained flies D. Biological processes downregulated in t_0 versus t_L condition in trained flies E. Biological processes upregulated in trained flies at t_L as a response to starvation relative to fed flies. F. Biological processes downregulated in trained flies at t_L as a response to starvation relative to fed flies.

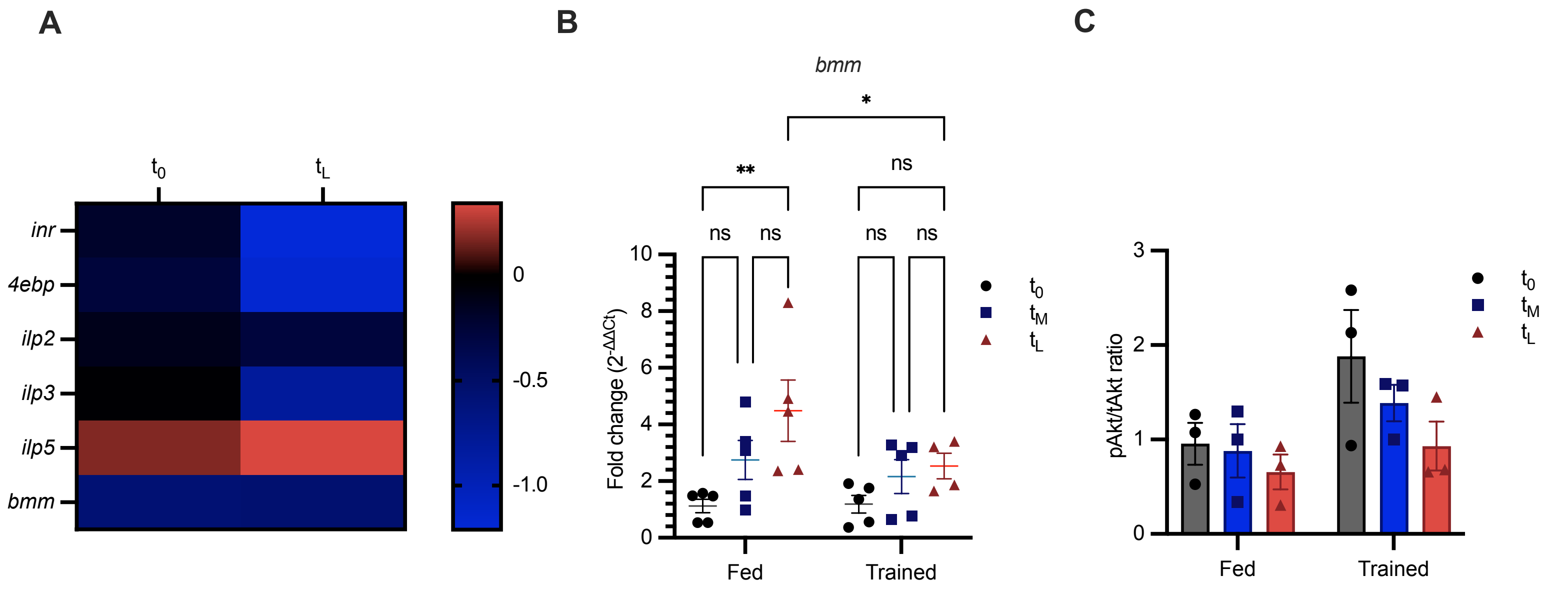

Fig. S3. Exposure to brief starvation modulates insulin responses of trained flies in response to starvation A. Heat map of insulin signalling-related gene expression in trained flies relative to fed control in response to starvation B. *brummer* transcript levels of fed and trained flies at t_0, t_M, and t_L *(fed t_0, t_M, t_L: n = 5, trained t0 and t_M: n = 5, trained t_L: n = 4)* C. Quantification of pAkt/tAkt ratio in fed and trained flies at t_0, t_M, and t_L *(n = 3) [B* and *C were analyzed with 2-way ANOVA with Tukey's HSD post-hoc test for multiple comparisons. p-value *<0.05; ** <0.01,*** <0.001, **** <0.0001. Data is presented as mean ± SEM]*

Journal of Experimental Biology • Supplementary information

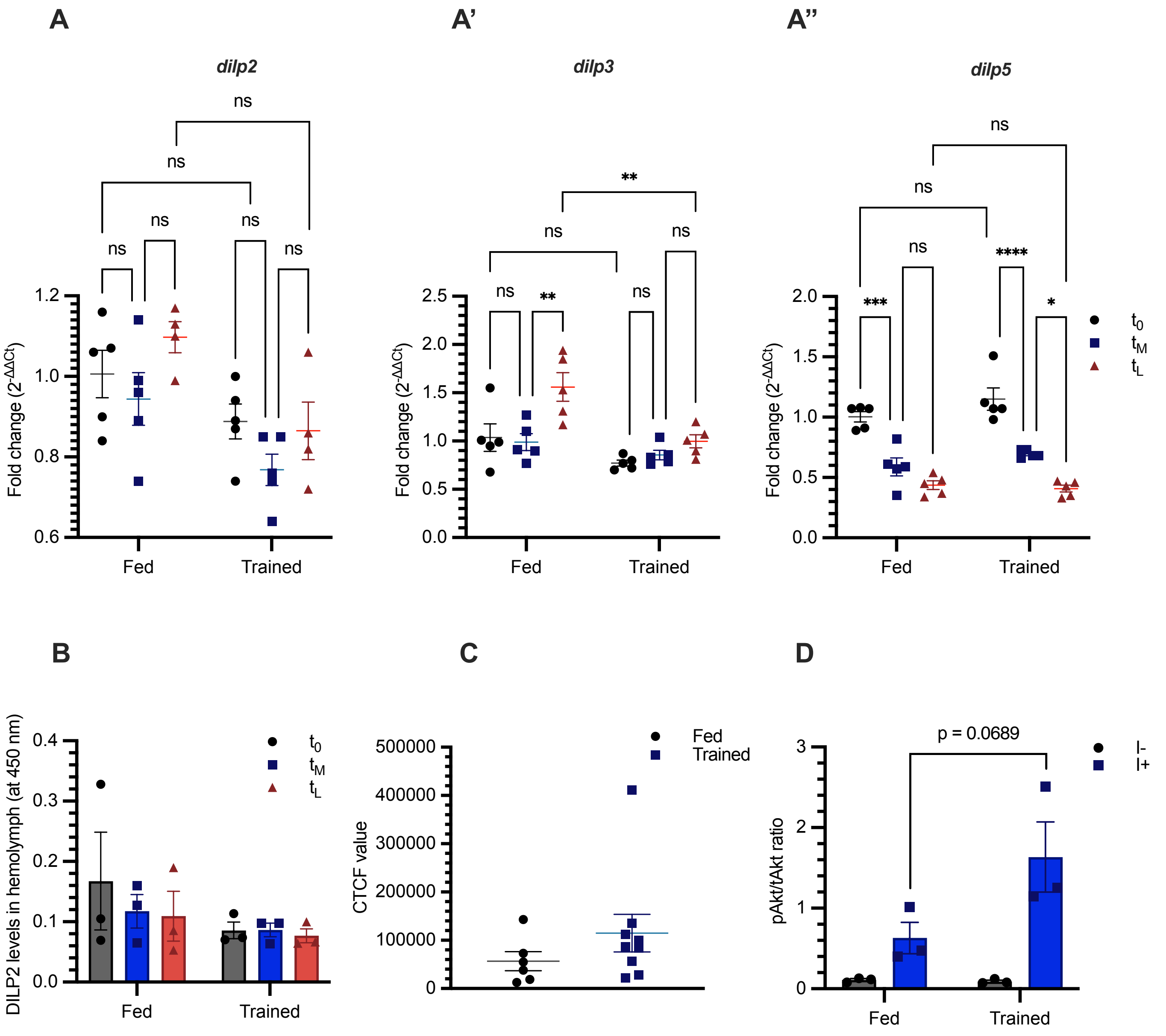

Fig. S4. Trained flies show no change in *dilp* expression but are insulin sensitive A. *dilp2* transcript levels *(n = 5 for all groups except t_L trained, where n = 4)* A’. *dilp3* transcript levels *(n = 5)* A’’. *dilp5* transcript levels *(n = 5)* at t_0, t_M, and t_L B. DILP2 levels in the hemolymph *(n = 3)* C. Corrected total cell fluorescence (CTCF) of DILP2 in insulin producing neurons at t_0 *(fed: n = 6, trained: n = 9)* D. Quantification of pAkt/tAkt ratio in fed and trained flies before and after insulin induction *(n = 3) [A, A’, A’’, B, and D were analyzed with 2-way ANOVA with Tukey’s HSD post-hoc test for multiple comparisons. C was analyzed with Mann-Whitney test. p-value *<0.05; ** <0.01,*** <0.001, **** <0.0001. Data is presented as mean ± SEM]*

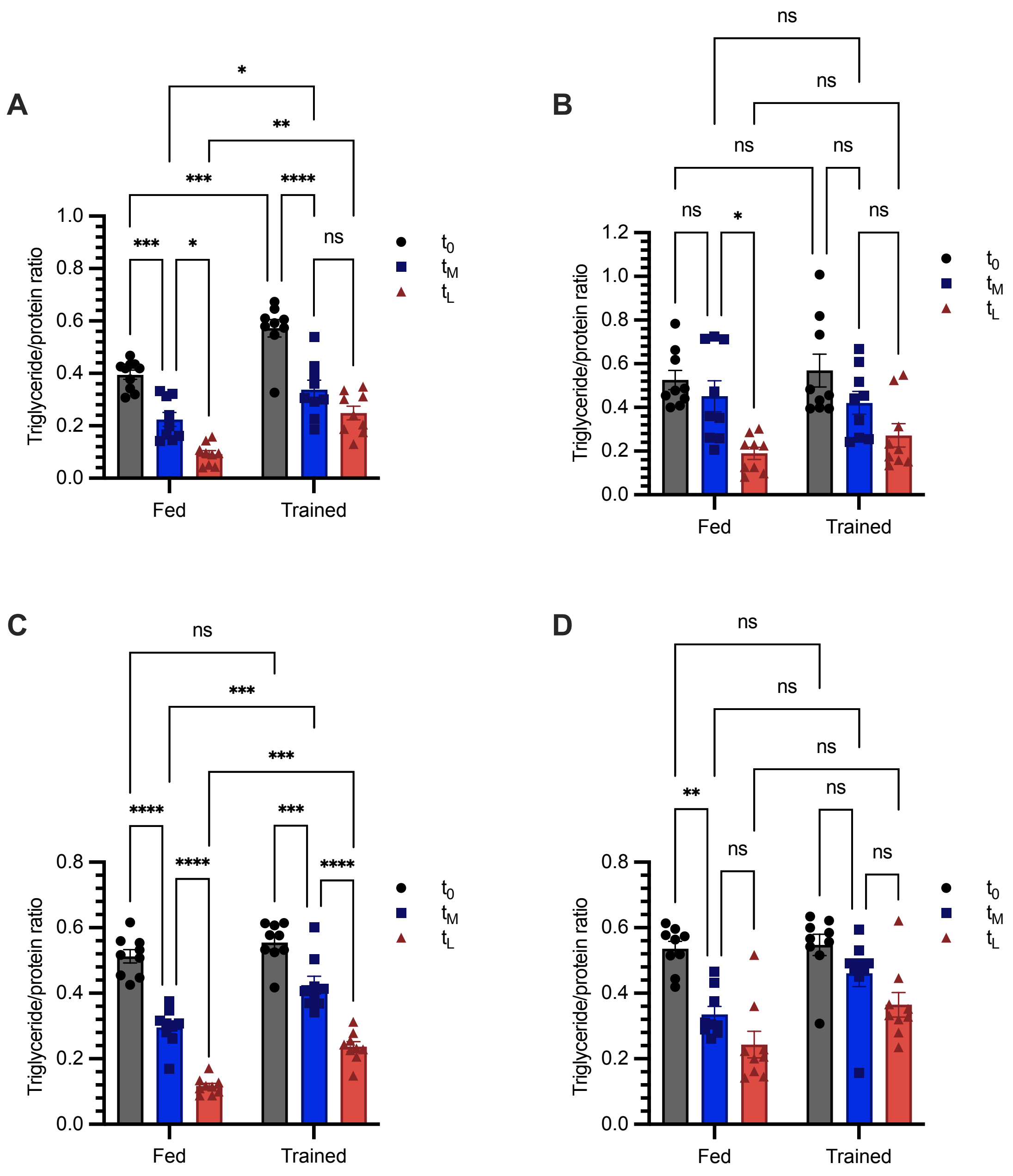

Fig. S5. Effect of blocking of IPC-derived and fat body-specific insulin signaling on triglyceride levels A. Triglyceride/protein ratio of *d2g4gs>w[1118]* flies at t_0, t_M, and t_L time points *(n = 9 for all groups except t_0 fed, where n = 10)* B. Triglyceride/protein ratio of *d2g4gs>kir2.1* flies at t_0, t_M, and t_L time points *(n = 9)* C. Triglyceride/protein ratio of *$s_1$106g4>w[1118]* flies at t_0, t_M, and t_L time points *(n = 9)* D. Triglyceride/protein ratio of *$s_1$106g4>inr-rnai* flies at t_0, t_M, and t_L time points *(n = 9) [Data was analyzed with 2-way ANOVA with Tukey's HSD post-hoc test for multiple comparisons. p-value *<0.05; ** <0.01,*** <0.001, **** <0.0001. Data is presented as mean ± SEM].*

Journal of Experimental Biology • Supplementary information

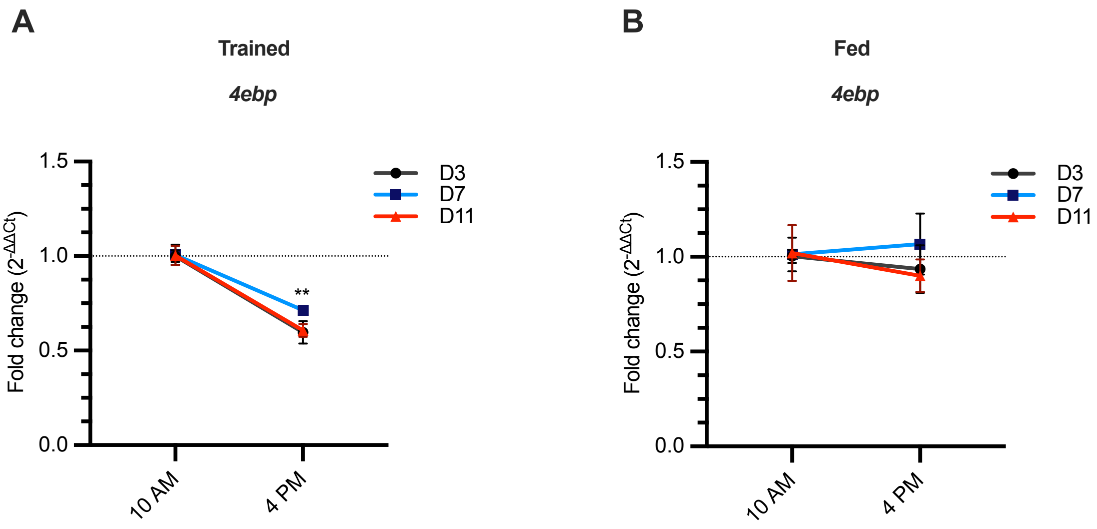

Fig. S6. Cyclical fed-fast regime leads to cycling of *4ebp* in trained flies A. *4ebp* transcript levels of trained flies at 10 am and 4 pm time points on day 3, day 7, and day 11 of training *(n = 3)* B. *4ebp* transcript levels of fed flies at 10 am and 4 pm time points on day 3, day 7, and day 11 *(n = 4 for all groups except D11 fed and trained, where n = 3) [Data was analyzed with 2-way ANOVA with Tukey's HSD post-hoc test for multiple comparisons. p-value *<0.05; ** <0.01,*** <0.001, **** <0.0001. Data is presented as mean ± SEM].*

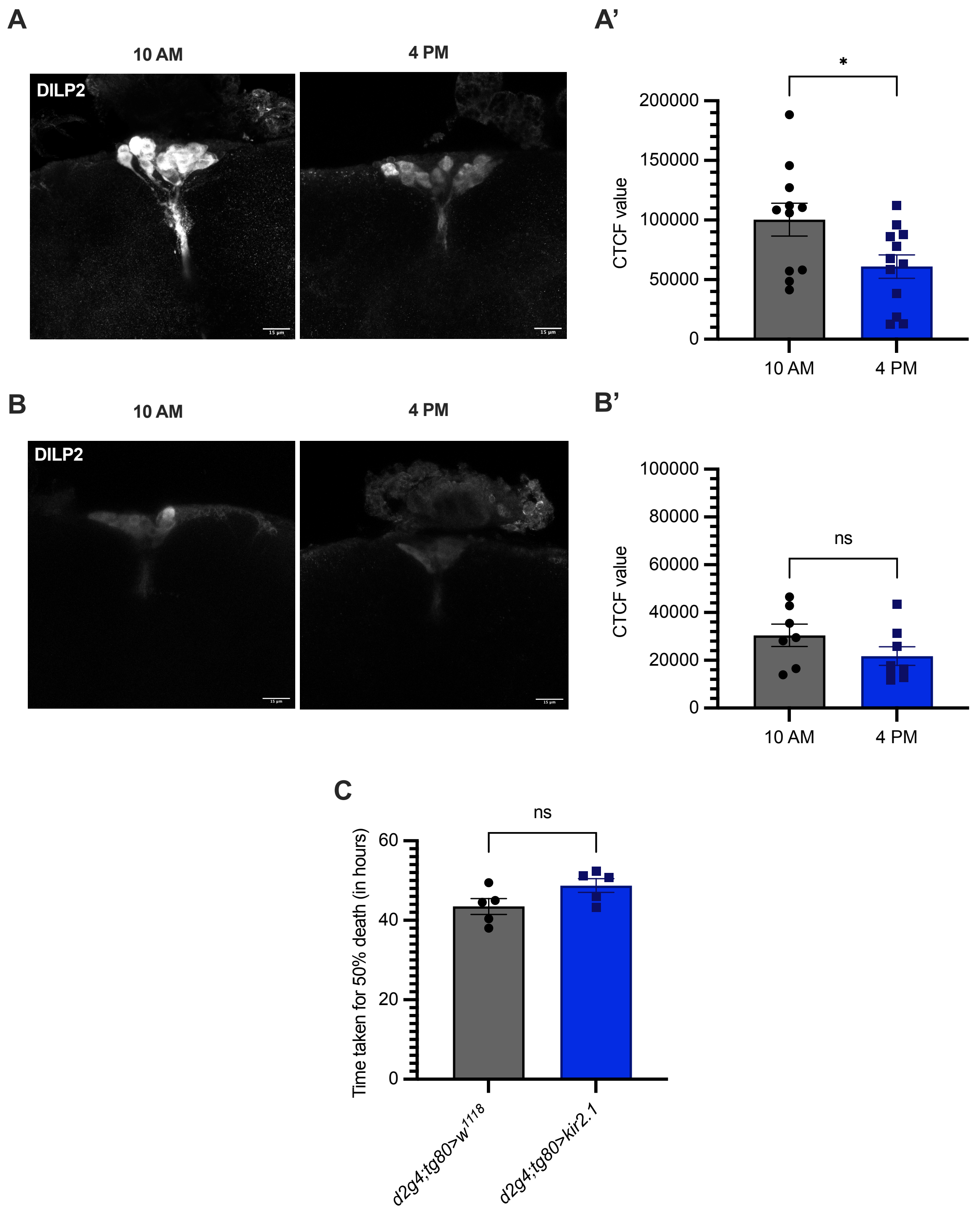

Fig. S7. A. DILP2 staining of the IPCs of *d2g4;tg80>kir2.1* flies at 10 am and 4 pm before switching to 18°C and 29°C respectively. A'. CTCF quantification of DILP2 within the IPCs of *d2g4;tg80>kir2.1* flies at 10 am and 4 pm before switching to 18°C and 29°C respectively *(10 AM n = 11, 4 PM n = 12)* B. DILP2 staining of the IPCs of *d2g4;tg80>w^{1118}* flies at 10 am and 4 pm before switching to 18°C and 29°C respectively. B'. CTCF quantification of DILP2 within the IPCs of *d2g4;tg80>w^{1118}* flies at 10 am and 4 pm before switching to 18°C and 29°C respectively *(10 AM n = 7, 4 PM n = 8)* C. Median survivorship of *d2g4;tg80>w^{1118}* and *d2g4;tg80>kir2.1* flies at 18°C *(n = 5) [Data was analyzed using Student's t-test with Welch's correction. p-value *<0.05; ** <0.01,*** <0.001, **** <0.0001. Data is presented as mean ± SEM].*

Table S1A. List of primers used

Gene name	Forward primer	Reverse primer
rp49	GCTAAGCTGTCGCACAAA	TCCGGTGGGCAGCATGTG
4ebp	CACTCCTGGAGGCACCA	GAGTTCCCCTCAGCAAGCAA
inr	AACAGTGGCGGATTCGGTT	TACTCGGAGCATTGGAGGCAT
dilp2	GGCCAGCTCCACAGTGAAGT	TCGCTGTCGGCACCGGGCAT
dilp3	CCAGGCCACCATGAAGTTGT	TTGAAGTTCACGGGGTCCAA
dilp5	TCCGCCCAGGCCGCAAACTC	TAATCGAATAGGCCCAAGGT

Table S1B. List of antibodies used and references

Antibody	Reference
#4060, Cell Signaling Technology	Slack et al., 2015, Liu et al., 2022
#9272, Cell Signaling Technology	Liu et al., 2023
#612656, BD Biosciences	Pathak and Varghese, 2021
#7074, Cell Signaling Technology	Lennicke et al., 2025
#7076, Cell Signaling Technology	Lennicke et al., 2025
DILP2	Sudhakar et al., 2020

Table S2. Genes involved in trehalose and glycogen synthesis upregulated in trained flies in comparison to fed flies post starvation

Gene name	log2FC	padj
tps1 (Trehalose-6-phosphate synthase 1)	1.17	2.56E-13
gbs-70e (Glycogen binding subunit 70E)	2.18	1.81E-47
ugp (UDP-glucose pyrophosphorylase)	1.92	3.33E-18
pgm1 (Phosphoglucomutase 1)	1.49	1.13E-18

Journal of Experimental Biology • Supplementary information

SUPPLEMENTARY REFERENCE LIST

1. **Lennicke, C., Bjedov, I., Grönke, S., Menger, K. E., James, A. M., Castillo-Quan, J. I., van Leeuwen, L. A. G., Foley, A., Buricova, M., Adcott, J., et al.** (2025). Enhancing autophagy by redox regulation extends lifespan in Drosophila. *Nat Commun* **16**, 5379.
2. **Liu, P., Chang, K., Requejo, G. and Bai, H.** (2022). mTORC2 protects the heart from high-fat diet-induced cardiomyopathy through mitochondrial fission in Drosophila. *Front Cell Dev Biol* **10**, 866210.
3. **Liu, J., Zhang, Y., Wang, Q.-Q., Zhou, Y. and Liu, J.-L.** (2023). Fat body-specific reduction of CTPS alleviates HFD-induced obesity. *Elife* **12**, e85293.
4. **Pathak, H. and Varghese, J.** (2021). Edem1 activity in the fat body regulates insulin signalling and metabolic homeostasis in Drosophila. *Life Sci Alliance* **4**, e202101079.
5. **Slack, C., Alic, N., Foley, A., Cabecinha, M., Hoddinott, M. P. and Partridge, L.** (2015). The Ras-Erk-ETS-Signaling Pathway Is a Drug Target for Longevity. *Cell* **162**, 72–83.
6. **Sudhakar, S. R., Pathak, H., Rehman, N., Fernandes, J., Vishnu, S. and Varghese, J.** (2020). Insulin signalling elicits hunger-induced feeding in Drosophila. *Developmental Biology* **459**, 87–99.

Journal of Experimental Biology • Supplementary information
